# A comparative study of earthquake disaster management laws between USA and Indonesia

**DOI:** 10.4102/jamba.v16i1.1582

**Published:** 2024-02-22

**Authors:** Ardianto B. Rahmawan, Gabriela Eliana, Latif A. Habibi, Alyca A. Nariswari

**Affiliations:** 1 Department of Administrative Law, Faculty of Law, University of Gadjah Mada, Yogyakarta, Indonesia; 2 Department of Business Law, Faculty of Law, University of Gadjah Mada, Yogyakarta, Indonesia; 3 Department of Civil Law, Faculty of Law, University of Gadjah Mada, Yogyakarta, Indonesia

**Keywords:** Indonesia, earthquakes, disaster management, agencies, regulatory problems

## Abstract

The frequent occurrence of disasters because of Indonesia’s geographical location within the Ring of Fire calls for effective disaster-management efforts. With the issuance of Law No. 24 of 2007 on Disaster Management, the Indonesian National Disaster Management Agency (Badan Nasional Penanggulangan Bencana or BNPB) was subsequently established. Keeping in mind previous disasters in Indonesia which occur in different regions, that is, Palu and Aceh Tsunami, Cianjur earthquake, and Mount Merapi, Indonesia’s disaster-management law is general and lacks technical guidelines in addressing the needs of the various regions and the different contours of geography. This article aims to analyse existing Indonesian regulatory and agency problems in disaster management by conducting a normative and comparative approach between the disaster management policy in the United States of America (USA) and Indonesia. The authors chose the USA’s disaster-management policy in particular as most comparative studies on disaster management law do not involve the USA despite the State’s ability to provide a comprehensive and consistent policy in the midst of the decentralisation of responsibilities with the numerous states. The authors conclude that according to existing disaster-management laws in USA and Indonesia, the government is obligated to fulfil its citizens’ rights in the event of natural disasters. Furthermore, noting the issues that exist within the Indonesian disaster-management system, Indonesia can adopt the best practices of the USA to improve its disaster-management system. Among others, Indonesia can learn from the USA in the fields of accountability and transparency amongst agencies, the development of Early Warning Systems, an improved system of interagency cooperation, as well as the provision of specific disaster-management assistance.

**Contribution:**

The findings of this study are expected to serve as evaluation material and to improve government effectiveness in dealing with natural disasters.

## Introduction

Natural disasters are a global concern for human sustainability. The complexities and unpredictability of disasters call for a cohesive disaster-management system (Lin 2018). Disaster management involves a systematic process of institution administration integrating competencies and strategic policies to reduce disaster hazards (Lin 2018). Commonly, disaster management requires institutional integrations consisting of public, private, and philanthropic institutions that respond to risks through improved strategic risk management, which work closely in the preparedness, mitigation, and prevention stages.

In facing the challenges of disasters, governments must coordinate effectively and take precautions. However, the extent of a government’s liability might be questionable. By analysing regulations and cases of disaster management, this article intends to unfold the liability of government agencies from disasters.

The Indonesian population, comprising over 273.8 million people, faces a high number of disaster risks. With 128 active volcanic mountains, the majority of seismic activities are triggered by complicated tectonics. Few outcomes of volcanic eruptions are largely known and predictions are improving with the advancement of technologies, yet most of the time, citizens must be prepared to evacuate and recovery may be unclear (Faure & Wibisana 2013).

According to the government’s estimation, 97% of the Indonesian population lives in disaster-prone areas, where earthquakes are amongst the highest risk; 62.4% of the Indonesian population is exposed to earthquakes (Nugroho, Ritonga & Anggraini 2015). To optimise the victims and adverse effects of a disaster, it can be measured by the risk and consequences of a disaster risk assessment formula, namely Risk = Hazard × Vulnerability/Capacity (Sari & Innaqa 2017). The Indonesian government uses this formula to measure the risk of a disaster and allocate disaster-related budgets (Haris et al. 2023).

Article 5 of Law No. 24 of 2007 on Disaster Management (‘*Indonesian Disaster Management Law*’) mandates the government and regional government to be responsible for disaster management. Under the law, Indonesian citizens are entitled to the rights to social protection and security, the fulfilment of basic needs (i.e. clean water, food, clothing, health services, and shelter). Thus, the state must ensure the citizens’ well-being, including the fulfilment of their rights post-disasters.

Domestically, the BNPB is the primary organisation charged with commanding, coordinating, and executing catastrophe risk cycles for the local disaster-management authorities (*Badan Penanggulangan Bencana Daerah* or ‘*BPBD*’) at provincial, regency, and municipal levels. There are several problems associated with the BNPB, that is, budget-related matters, inter-agency cooperations, and the lack of disaster management standard operating procedures (‘*SOP*’) (Tanesab 2020). Failure to resolve these issues would affect disaster-management efforts conducted by the agency, possibly depriving citizens of their rights to receive disaster-management support from the government. Accordingly, efforts must be made to solve such issues.

Conversely, the US also experiences natural disasters such as earthquakes, volcanic eruptions, floods, droughts, and mudflows. The disaster-management responses in the U.S. are structured using several layers comprising District, State, and Federal levels; the split is mainly the result of the U.S. federal structure. The U.S. Constitution specifies power retained by the State and power transferred to the federal government. Hence, despite the statutory split preserving and delineating State and Federal authority, joint action and uniformity are possible. Disaster management in the U.S. necessitates the coordination of multiple agencies ranging from local police, fire, and health departments to the Federal Emergency Management Agency (‘*FEMA*’) (FEMA 2023). The U.S. disaster management focuses on four important areas: mitigation, training, response, and recovery (FEMA 2023). Disaster-management efforts of incidents exceeding the state’s capacity to respond and recover will be supported by the federal government. The Federal government has been primarily responsible for promoting greater standards across the nation and providing substantial financing for mitigation, training, and management (La Union Del Entero v. Fema 2010; St. Tammany Parish ex rel 2009, Davis v. FEMA v. Federal Emergency Management).

Upon understanding the U.S. and Indonesian disaster management, it would be beneficial if some of the practices in the U.S. were to be implemented domestically. This is because *firstly*, the U.S. has been able to demonstrate effective inter-agency cooperation, whereas Indonesia also requires cooperation with numerous agencies during disasters. *Secondly*, the U.S. has been able to conduct effective disaster-management efforts despite the numerous federal states with different needs. Similarly, Indonesia has numerous provinces with differing disaster risks and post-disaster recovery needs. *Finally*, the U.S. government is not statutorily required to provide governmental assistance during and after disasters, while Indonesia imposes that the government is automatically responsible to conduct disaster-management efforts and provide assistance.

## Research methods and design

In conducting this research, the authors resorted to a normative research in a qualitative manner. In analysing the different disaster-management regulations in Indonesia and the U.S., the authors adopted a comparative approach. With the aforementioned approaches, the author aims to identify the concepts of liability and the expected conduct of agencies in the fields of disaster management in the different jurisdictions, as well as to identify the best practices of the U.S. in regulating its disaster-management system to allow the authors in providing recommendations to Indonesia. Moreover, in analysing the different practices of existing disaster-management regulations in Indonesia and the U.S., the authors utilised a case law analysis on disaster management-related cases in the said jurisdictions.

The authors resorted to primary sources including laws relevant to disaster management in Indonesia and the U.S. To interpret such primary sources, the author referred to secondary sources in the form of journal articles, books, conference papers, as well as court decisions. These sources are utilised by the authors to analyse the indicators of regulatory and practical aspects of the disaster-management system in Indonesia and the U.S.

### Ethical considerations

This article followed all ethical standards for research without direct contact with human or animal subjects.

## Discussion and analysis

### State’s roles in responding to disasters

#### Government’s mandate to execute disaster-management response

A universal problem amongst countries is natural disasters, which is known to frequently cause casualties and losses. The Inter-Agency Security Committee (‘*IASC*’), a body overseen by the UN, was created in response to this, establishing executive guidelines with international non-governmental organisations (‘*NGOs*’) that include disaster-management principles. The guidance essentially lists the fundamental rights that States must uphold in natural disasters, including the right to life, guarantees of evacuation, relocation, and other rescue measures (IASC 2006).

Asian countries share a high exposure to natural and human-induced risks but each country’s disaster-management system effectiveness varies (Cook & Dorussen 2021). Foremost, Indonesia faces an increasing level of disaster risk. With Indonesia’s location in Southeast Asia, Indonesia is prone to natural disasters, which makes Indonesia vulnerable to adverse effects if disaster strikes (Asian Development Bank 2021). Accordingly, the Indonesian government guarantees its citizens protection through the explanation of Indonesian Disaster Management Law and Paragraph IV of the Indonesian Constitution. Furthermore, in the Indonesian Disaster Management Law, the state is responsible for preventing, responding, and recovering from natural disasters. Indonesia recognises at least three types of disasters, namely natural, non-natural, and social disasters (Indonesian Disaster Management Law 2007). It is explicitly stated that natural disasters are among the types of disasters that are fully covered by the government’s responsibility.

The Indonesian government is responsible for disaster-management implementation, that is, disaster risk reduction, integration with development programs, disaster protection, fair and equitable realisation of communities’ and refugees’ rights, disaster recovery, budgets-related obligations, and keeping trustworthy archives of disaster threats and impacts (Indonesian Disaster Management Law 2007).

The Indonesian Disaster Management Law essentially provides the obligations that the government has to fulfil in managing disasters as well as citizens’ rights that are related to disaster management. However, as the law does not provide specific technical guidelines tailored to the different contours of geography in the various regions of Indonesia, there is certainly room for improvement in the law. The enforcement of these rules has also been outlawed in some cases where the government provides compensation for damages or other forms of responsibility to victims of natural disasters. One such instance was the 2017 Mampang River Flood. Victims of the natural disaster filed a complaint against the government for failing to take precautionary steps, which resulted in the flooding. The judge granted the lawsuit in the verdict, and the government is required to rehabilitate the Mampang River construction (Tri Andarsanti et al. 2022, v. Gubernur Provinsi DKI Jakarta, 205/G/TF/2021/PTUN.JKT).

#### Types of liabilities

Liability is a framework of government accountability designed to protect its citizens’ human rights. Liability enforcement is an expression of the rule of law notion and an acceleration of good governance (Jiwantara, Dewi & Supryadi 2022). There are various factors that can result in agencies being held liable, including negligence, intentional torts, vicarious liability, strict liability, and liability based on fault. However, only negligence, strict liability, and liability based on fault are commonly discussed in the context of natural disasters.

Negligence occurs when a subject causes injury to another person by failing to meet a legal duty to act with the required level of care; they have a standard of care to maintain. Accordingly, actions were taken regardless of the fact that they were unreasonably safe (Nicholson 2012). A victim, however, does not have to establish that a subject wilfully harmed them. Thus, parties can be held liable when actions were carelessly taken (Congressional Research Service 2023). Parties will have to show elements of duty, breach of duty, legal causation, personal injury or property damage, and result. Negligence in the wake of natural catastrophes generally results from the neglect of specific governmental obligations (Nicholson 2012). Failure to complete commonly acknowledged tasks as part of emergency management responsibilities is another frequent source of liability.

Strict liability is defined as establishing liability regardless of the defendant’s level of care. In general, the plaintiff does not need to prove the defendant’s culpability; only proof of loss and causality for the incident that occurred has to be proven (Congressional Research Service 2023). There is a common misperception about strict liability, which holds that the defendant bears no burden of proof. In reality, the defendant still bears the burden of proving the loss and cause.

Conversely, liability based on fault requires the plaintiff to prove the defendant’s culpability. In deciding liability based on fault, proving the element of the defendant’s fault is critical. Another type of liability is intentional tort, which requires the plaintiff to prove that the defendant deliberately intended to cause a disaster or loss. Table 1 indicates a comparison of agencies liability.

**TABLE 1:** Table comparison of agencies liability.

Comparition	U.S.	Indonesia
**Definition of disasters/ Acts of God**	Any natural catastrophe or any fire, flood, or explosion regardless of its cause; Considered by the U.S. President to have caused damages of sufficient severity and magnitude.	An event or series of events that disrupts society; Caused by natural, non-natural, or human factors; Resulting in human casualties, environmental damages, and property losses.
	Natural phenomena that are grave; Exceptional, inevitable, and irresistible; Causing effects that could not have been prevented even after the exercise of due care or foresight.	
**Type of liability**	Strict liability Negligence	Strict liability

#### Applicable liability toward governments in disaster management

The concept of liability towards governments varies depending on each country’s regulations. Several countries adopt an approach recognising sovereign immunity towards the government, while others uphold that the government can be held liable and is not immune from civil or tort suits. In the U.S., the doctrine of state sovereign immunity is waived by the *Federal Tort Claims Act*, while in Indonesia, government agencies may be held liable for violations of the law as exemplified by the numerous class actions and proceedings in administrative courts towards governmental agencies (Nugroho, Harjiyatni & Rahardja 2020). Accordingly, both Indonesia and the U.S. welcome the possibility of governmental agencies being held liable for violations of the law, which may inevitably include disaster management-related violations.

Upon establishing the concept of liability and acknowledging the existence of sovereign immunity towards the government, it would be important to draw the lines of what constitutes *force majeure* events. Despite the possibility of governmental agencies being held liable for disaster management-related and environmental law-related cases (Faure & Wibisana 2013), whether or not disasters are *force majeure* may also determine the government’s liability.

The U.S. *Comprehensive Environmental Response Compensation and Liability Act* (‘*CERCLA*’) defines ‘act of God’ as natural disasters or phenomenon that are grave, possessing the characters of being exceptional, inevitable, and irresistible, and causing effects that could not have been prevented even after parties have exercised due care or foresight. Where a phenomenon constitutes an act of God, the U.S. government will be liable to provide environmental recovery assistance (Cercla 1980; Sabine Towing & Transportation Co. v. United States 1981). Moreover, the *Stafford Act* provides that a ‘major disaster’ is any natural catastrophe or any fire, flood, or explosion regardless of its cause, in any part of the U.S., which is determined by the U.S. President to be a phenomenon causing damages of sufficient severity and magnitude.

The practice in the U.S., as reflected by *Sabine Towing*, reflects that the elements of exceptional, inevitable, and irresistible, must be proven to categorise the phenomenon as an act of God. Failure to prove so will render that the government is liable to provide assistance (Faure & Wibisana 2013). In *Sabine Towing*, disasters must also ‘be of great magnitude’, thus emphasising the importance of the gravity of disasters. The case of Joseph Resnick, Co., Inc. (1963) further highlights that acts of God can be proven if there is no human intervention or reasonable capacity that can prevent the disaster from occurring (Faure & Wibisana 2013).

Conversely, in Indonesia, liability for disaster management immediately falls on the government as reflected under the Indonesian Disaster Management Law and Indonesian Law No. 29 Year 2014 on Search and Rescue. Thus, so long as a phenomenon is categorised as a disaster, the government has the responsibility to conduct disaster-management efforts. In determining whether a particular phenomenon is a disaster, the Disaster Management Law upholds that it must be: (1) an event or series of events that disrupt the society, (2) caused by either natural, non-natural, or human factors, (3) which results in human casualties, environmental damages, property losses, and psychological impacts. A phenomenon will generally be classified as a disaster if it was an inevitable phenomenon causing severe damage to the society that could not have been prevented, regardless of whether it was natural or non-natural.

Though Indonesia is not constricted to precedents, previous disaster-related cases provide enlightenment on Indonesian practices. Constitutional Review No. 83/PUU-XI/2013 regarding Lapindo Victims exhibits that current practices are in line with the statutory provision under the Indonesian Disaster Management Law. *In casu*, the government is responsible for carrying out disaster-management efforts (Constitutional Court No. 83/PUU-XI/2013 2014). Additionally, the 2022 Palembang Flood Class Action under Case No. 10/G/TF/2022/PTUN.PLG, aside from proving that court judgments are in line with the above law, reflects that the government is liable for post-disaster management and disaster prevention (Ali et al. 2022, v.Walikota Palembang).

Based on the preceding assessment, strict responsibility appears to be the most suited for natural catastrophes since natural disasters require a different approach as their phenomena are impossible to forecast. Therefore, the concept of liability must be explicit in terms of proof. In comparison to negligence, vicarious liability, and intentional tort, strict liability has the advantage of not requiring proof of error. In the event of natural disasters, the process is critical for citizens to claim the government’s duty if sufficient attempts were not made to protect citizens.

Regardless of the above, in 2012, the Committee on Guidance for Establishing Crisis Standards of Care for Use in Disaster Situations of the U.S. Institute of Medicine issued guidance on standards of care and legal liability in response to the enormous amount of civil suits against emergency medical personnel following the 2005 Hurricane Katrina response. The Crisis Standards of Care essentially highlights the possibility of liability protection. Such liability protections can also be seen under other regulations including the *Stafford Act* and CERCLA (Altevogt et al. 2009).

## Indonesia on disaster management

### Regulation

Disaster-management provisions are mainly upheld under the Indonesian Disaster Management Law, comprehensively regulating pre- to post-disaster management, division of responsibilities between governments, establishment of disaster institutions, and involvement of NGOs. The law also forms the basis of the Government’s responsibility on disaster management, at the same time allowing decentralisation as exemplified by the establishment of BNPB and BPBD; such decentralisation effort is partially implemented (Das & Luthfi 2017).

The Indonesian Disaster Management Law causes the establishment of numerous regulations related to disaster management, including governmental regulations. Other disaster-related provisions are contained in various laws and regulations as reflected in Table 2.

**TABLE 2:** Related-regulation on disaster risk management.

Year	Disaster risk management-related law
2004	Law 25/2004 on National Development Planning System
2007	**Law 24/2007 on Disaster Management (DM)** Law 26/2007 on Spatial Planning Law 27/2007 on the Management of Coastal Areas and Small Islands
2008	Government Regulations (‘**GR**’): ■ GR 21/2008 on Disaster Management; ■ GR 22/2008 on Disaster Management financing and aid assistance; and ■ GR 23/2008 on Disaster Management external supports (International agency and non-governmental agency); Presidential Regulation (Perpres) 8/2008 on the establishment of National Disaster Management Agency (BNPB); Minister Regulation of Ministry of Home Affairs (Permendagri) 46/2008 on Organizational and Management of Local Disaster Management Agency (BPBD); Regulation of the Head of BNPB (Peraturan Kepala BNPB) 3/2008 on the establishment of Local Disaster Management Agency (i.e. BPBD); ■ And many other regulations of the Head of Disaster Agencies or Ministers.
2009	Law 31/2009 on Meteorology, Climatology and Geophysics; and Law 32/2009 on the Protection and Environmental Management
2014	Law 23/2014 on the Regional Government; Law 6/2014 on Village
2016	Minister Regulation of Ministry of Energy and Mineral Resources (KESDM) 11/2016 on Geological Disaster Prone Areas

The Indonesian Disaster Management Law specifies the government’s pre-disaster management responsibilities, including the mitigation of disasters through spatial planning, constructing facilities, and education. In the post-disaster sections, Article 26 provides that citizens affected by disasters are entitled to receiving disaster reliefs. Such reliefs may include clean water and sanitation, food, clothing, health services, psychosocial services, and shelter-related needs, as well as compensation for the death of individuals and disabilities caused by the disaster. Paragraph 3 related to post-disasters regulates the possibility of rehabilitation and reconstruction assistance, which includes the provision of help towards repairment of housings and public facilities, as well as the reconstruction of facilities.

#### General overview of disaster-management practice in Indonesia

The Indonesian Disaster Management Law aims to establish a systematic disaster-management framework for Indonesia, especially noting previous catastrophic disasters (e.g. 2004 Aceh Tsunami and 2006 Jogja Earthquake). Prior to this law, disaster-management efforts were focused on establishing ad-hoc institutions post-disasters. This is exemplified by the NAD-Nias Rehabilitation and Reconstruction Agency, an ad-hoc institution established shortly after the 2004 Aceh Tsunami (Mardiah, Lovett & Evanty 2017). The aforesaid law thus shifts the disaster-management paradigm from reactive to preventive.

At the central level, the government’s responsibilities include disaster risk reduction (DRR) integrated with national development, community protection against disasters, and disaster-management budget allocation (Indonesian Disaster Management Law 2007). The BNPB has a ministerial-level position and is directly responsible to the President, where the institution is authorised to lead the coordination and consolidation of disaster management with the government and relevant institutions. The institution complies with the Indonesian Disaster Risk Index (BNPB 2022), which acts as the government’s basis in formulating disaster-management strategies as outlined in the National Medium-Term Development Plan which is issued by the government every 5 years, and acts as the basis for the National Regional Spatial Plan.

Within the regional level, key disaster-management agencies are Regional Disaster Management Agencies or BPBD and Provincial Development Planning Agency or Bappeda. Both actors are responsible for internalising the DRR agenda into regional development policies, that is, the Regional Medium Term Development Plan. The BPBDs have three functions. Firstly, the coordination function as per Head of BNPB Regulation No. 3 of 2008, where the BPBD is at the forefront of communicating about disaster conditions towards other levels of the government. Secondly, the command function, which is interconnected with disaster emergency status. Finally, the control function, which is linked to early warning systems (EWS) utilisation, disaster risk-based spatial planning, and the determination of Disaster-Prone Areas.

#### The lack of coordination between institutions

BNPB plays a crucial role as a coordinator in establishing and implementing disaster risk reduction (‘*DRR*’) efforts (Tanesab 2020). There are seven key players in the DRR agenda: BNPB, the MoHA, the Ministry of Public Work and Housing, BPBDs, BAPPEDAs, NGOs, and Universities (Mardiah et al. 2017). Apart from BNPB and the said institutions, Indonesia has other agencies working in the disaster sector which provide innovation and coordinate early warning systems (‘*EWS*’) such as BMKG, Geological Agency, and BPPT (now merged to National Research Agency). Those agencies will mine and gather data to be used by BNPB to plan disaster-management efforts. Disaster institutions have developed technology-based disaster-management equipment and infrastructures which play a crucial role in disaster management. However, despite BNPB leading several institutions, there is no clear chain of command (Meilani & Hardjosoekarto 2020). Moreover, those agencies develop their Disaster EWS without clear coordination with BNPB or other institutions (Moorthy, Benny & Gill 2018). Such competition from agencies may create misinformation especially when they are not interconnected (Meilani & Hardjosoekarto 2020).

Since the Aceh Tsunami in 2004, disaster communication in Indonesia needs to be improved to reduce the number of losses (Meilani & Hardjosoekarto 2020). Disaster communication is crucial in every disaster-management stage: pre-disaster, during disasters, and post-disaster. At the pre-disaster stage, disaster outreach to the community and related institutions is crucial because it will increase community capacity, thereby reducing disaster risks (Sari & Innaqa 2017). However, socialisation of earthquakes and development of technology to predict natural disasters in Indonesia seems to be minimal (Hidayat 2023). Communication during and after disasters is also vital; accurate information during and after disasters must be disseminated vastly.

Currently, BMKG’s ‘InfoBMKG’ mobile application has a high level of speed and accuracy in disseminating information to the public. However, inter-agency communication and communication with the public still needs to be improved, especially noting that the InfoBMKG application is still under the Beta version; poor communication frequently causes casualties.

Spatial planning also plays a crucial role in minimising harms and losses post-disasters. The Indonesian spatial planning system is regulated under Law No. 26 of 2007 on Spatial Planning and its implementing regulations, where such regulations have considered spatial planning in conjunction with disaster management. However, in practice, the government’s supervision of spatial use is still lacking. In Bandung, West Java, insufficient local government oversight of spatial use has resulted in earthquake-prone areas being converted into new residential areas. In fact, Bandung is the home of 8.6 million people, where several citizens live on the soft-sediments of Bandung lake connected to the Lembang fault which is a potential earthquake source (Daryono et al. 2019). Similar conversion of land into housing on unstrategic locations also took place on the slopes of Mount Manglayang, Bandung, as well as Pleret, Bantul; the latter involves the massive conversion of agricultural land into houses when Pleret is in fact a zone with high vulnerability to earthquakes (Haryana, Fikriyah & Yulianti 2013; Regina Cantika, Asdak & Amaru 2019).

The aforementioned poor spatial planning is because of insufficient research efforts. In Palu, Central Sulawesi, the lack of spatial planning and disaster-management research results in the establishment of National Urban Development projects on soil liquefaction-prone areas, that is, Perumnas Balaroa (Ratode, Nugroho & Sufyandi 2021). Consequently, when a 7.5 SR earthquake hit Palu, causing soil liquefaction, Balaroa became the location mostly affected by such liquefaction which contributes to the huge casualties and material losses (Sagala et al. 2021). Such occurrences exemplify the lack of effective inter-agency cooperation in conducting spatial planning based on disaster-related risks.

The Sunda Strait Tsunami in December 2018 is evidence of poor communication between agencies. *In casu*, BNPB failed to immediately coordinate with other agencies on tidal waves (BNPB 2018). Meanwhile, BMKG noted an increase of abnormal sea waves along the Sunda Strait Coast, and the PVMBG further mentioned that there had been an increase in Anak Krakatau volcanic activity which might lead to Tsunami (Pelupessy et al. 2021). Such poor coordination led to massive damage and many casualties, as the Sunda Strait Tsunami caused the death of 437 people and injured 14.000 others (Dewi 2021).

#### Limited budget allocation

The central and regional governments are responsible to allocate disaster-management budgets as regulated under the Indonesian Disaster Management Law. Further provisions regarding budget allocation are also regulated in Government Regulation No. 22 of 2008 Disaster Relief Funding and Management. However, there are no specific regulations regarding the allocation of disaster-management funds under the State Budget. Consequently, disaster-management funds are not amongst the priorities of the State Budget plan.

Data compiled by the Ministry of Finance reflect that the average allocation for disaster reserve funds was Rp. 3.1 trillion in the period 2005–2015 (Aldin 2021). The average disaster budget allocation was less than 1% of the National Expenditure Budget, which also happened in previous years; disaster-management budgets in regions are also quite limited (Waneza, 2018). Disaster-management budgets in cities also receive the same treatment, where the DKI Jakarta government allocates less than 1% of its budget towards disaster management (Intarti, Fitrinitia & Widyanto 2012). Aceh also sees the same problem despite it being a disaster-prone area (Fahlevi, Indriani & Oktari 2019). Table 3 exhibits the allocation of disaster-management budgets in 2015 (Ministry of Finance 2015).

**TABLE 3:** Budget allocation on disaster management (2015).

No.	Ministry or agency	Budget
1.	Ministry of Home Affairs	Rp. 66 950 000 000
2.	Ministry of Public Works and Housing	Rp. 168 207 634 000
3.	Ministry of Social Affairs	Rp. 235 189 850 000
4.	Coordinating Ministry for Human Development and Cultural Affairs Indonesia	Rp. 10 239 000 000
5.	Ministry Of Village, Development Of Disadvantage Region, and Transmigration	Rp. 18 078 500 000
6.	National Disaster Management Agency	Rp. 986 245 600 000
7.	Sidoarjo Mudflow Disaster Management Agency	Rp. 837 529 057 001

**Total**		**Rp. 2 322 439 641 000 001**

*Source:* Madjid, N.C., 2018, ‘Analisis metode penghitungan dan alokasi anggaran bencana alam’, in *Simposium Nasional Keuangan Negara*, p. 1056, November 14–15, Kementerian Keuangan

Minimal budgeting entails the lack of maintenance of Indonesian EWS equipment. This issue has been exemplified by the fatal tragedy that occurred during the 2018 Sunda Strait Tsunami where the malfunction of the tsunami detection device (buoy) caused hundreds of casualties and other material losses (Solihuddin et al. 2020). The buoy, which was also installed by BPPT in the Sunda Strait in 2006 has not been functioning for a prolonged period of time as it did not receive proper maintenance (Meilani & Hardjosoekarto 2020). The merger of the National Research Agency further aggravates the utilisation of disaster-management budgets. Since September 2022, all Buoys in Indonesia have died and have not sent any signals (Hidayat 2023).

#### Implications of failure to conduct effective disaster-management response in Indonesia

Article 5 of the Indonesian Disaster Management Law upholds the fact that the implementation of disaster management is the central and regional government’s responsibility, an order from the law to policymakers (Triyana 2013). Failure to conduct effective disaster-management results in the inability to protect citizens, which may entail the central and regional government being held accountable with criminal charges for inability to conduct disaster-management efforts as stipulated in Article 75 of the law.

Article 75 of the said law is a very broad provision and can be imposed on every stakeholder in charge of administering disaster management. Criminal sanctions are also regulated in Law No. 25 of 2009 on Public Services as disaster management falls under the scope of public services. However, a Supreme Court Regulation stipulated that acts against the law conducted by the government indicates the competence of the Administrative Court. These provisions are contradictory as Administrative Courts may not grant criminal sanctions since the court can deal only with cases of an administrative nature (Ordinance of The Supreme Court (Peraturan Mahkamah Agung/PERMA) No. 2 Year 2019).

Indonesian Law No. 9 of 2004 on Amendments to Law No. 5 of 1986 regarding Administrative Courts upholds that administrative claims can be filed by Indonesian citizens through administrative courts. Furthermore, as disaster management falls under the scope of public services, the government’s failure to implement effective disaster management allows citizens to sue the government through administrative court (Indonesia Public Services Law 2009). Law No. 32 of 2009 on the Environment also regulates the rights of citizens and environmental organisations to file a lawsuit against the government either individually, as a group, or as a class action if losses occur because of environmental damage.

In 2021, community groups in Jakarta filed a suit against the provincial government at the Administrative Court for accusations of negligence in carrying out flood-prevention efforts on the Mampang River; the claim against the government’s obligation to conduct river normalisation was granted. However, the claim regarding compensation to the community because of material losses experienced was not granted (Tri Andarsanti et al. 2022, v. Gubernur Provinsi DKI Jakarta, 205/G/TF/2021/PTUN.JKT).

Another example of citizens filing a lawsuit against the government also includes the class action filed by community and environmental groups in regards to the 2021 Palembang flood (Ali et al. 2022, v. Walikota Palembang). Citizens argued that the flood occurred as a result of spatial planning error by the government, resulting in floods arising in areas that are not prone to flooding. The court granted all of the citizens’ claims, including the order towards the Palembang government to comply with provisions regarding green areas which must be at least 30%, restoring the function of swamps and retention basins as flood control, accommodating flood disaster prevention such as providing EWS, and paying compensation to the plaintiffs.

The previously discussed precedents reflect the fact that existing laws and regulations still allow citizens to file a lawsuit against the government when losses occur because of natural disasters through the Administrative Court. However, citizens may still face obstacles upon the grant of their lawsuit because of the absence of strict sanctions against the government (Lumbanraja, Utama & Putrijanti 2019).

### US on disaster management

#### Regulation

In the U.S., disaster management is characterised by a decentralisation of responsibilities at the State, Federal, local, and tribal levels, with FEMA taking the main responsibility (Whalen 2009). Despite FEMA being the primary agency to perform disaster-management efforts, the agency still has to coordinate with numerous agencies and NGOs. The U.S. government’s conduct in preventing, preparing, and responding to disasters is regulated under the *Stafford Act* and the *Post-Katrina Reform Act*. The former establishes how disasters shall be declared, the government’s disaster-management programs (Lindsay 2021; Moss, Schellhamer & Berman 2009), and grants the Federal Disaster Recovery Coordinator the authority to respond to disasters (National Disaster Recovery Framework 2016). The latter articulates governmental institutions responsible for conducting disaster management-related activities, that is, FEMA and the Committee on Homeland Security and Governmental Affairs of the Senate (*Post-Katrina National Emergency Management Reform Act* 2006, as amended 2019).

Under the *Stafford Act*, the President is authorised to establish a disaster preparedness program, which typically involves the planning of mitigation, warning, training, evaluations, annual review of programs, and interagency coordination. The act also specifically regulates the issuance of disaster warnings. In the pre-disaster field, the *Stafford Act* provides the possibility of the President to provide hazard mitigation assistance. Furthermore, the President is obligated to construct a Federal interagency task force to coordinate regarding pre-disaster hazard mitigation programs (*Stafford Act* 2021).

In regards to disaster management, the U.S. President has the authority to enable any U.S. federal agencies to provide disaster management essential assistance, such as:

- Utilisation of Federal facilities, personnel, and other resources;
- Distribution of foods and consumables;
- Performance of services essential to save lives and preserve properties, public health, and safety, including debris removal, road clearance, and the demolition of buildings;
- Other contributions provided to the state, local governments, or even private non-profit organisations (*Stafford Act* 2021).

Despite the *Stafford Act* wording the above assistance as ‘Essential Assistance’, such reliefs are contingent upon the President’s discretion, as the Act stipulates that the President ‘may’ provide the assistance, indicating that such may not be necessary.

Aside from Section 403 on essential assistance provided to victims, the *Stafford Act* opens up the possibility of the government to provide hazard mitigation towards projects, including relocation, installation of debris traps, construction of drainage dips, and emergency spillways. Additionally, the *Stafford Act* recognises the possibility of the President to provide disaster relief to individuals, including debris removal assistance, housing assistance (in monetary form or the provision of temporary housing units), the reparation of infrastructure, and financial assistance for matters ranging from medical, child care, funeral, and personal property (*Stafford Act* 2021).

#### General overview of disaster-management practice in the U.S.

The U.S. highlights the importance of disaster preparedness plans for pre- and post-disaster occurrence involving all relevant federal agencies (*Stafford Act* 2021). Existing examples of plans that have been constructed include State-wide Communication Interoperability Plans, logistics and resource management plans, other pre-disaster recovery plans (National Disaster Recovery Framework 2016), and post-disaster recovery plans including the Iowa Storms and Floods 2008 Long-Term Recovery Plan (FEMA 2015).

Regarding the structure of disaster management-related groups amidst inter-agency cooperation, the U.S. Government adopts a common structure of the incident command system to allow a unified command during incidents. This structure applies to various entities, that is, law enforcement, public health, etc., where they must communicate agency-specific information to minimise information overlaps. Further, the U.S. came up with Incident Management Teams, groups that operate during incidents at local, regional, state, national, and tribal levels (National Incident Management System 2017).

Regarding earthquakes, the *Earthquake Hazards Reduction Act* 1977 establishes the Interagency Coordinating Committee on Earthquake Hazards Reduction led by the Director of the National Institute of Standards and Technology (*Earthquake hazards reduction act* 1977, as amended 2022). The committee is composed of the FEMA Administrator, the U.S. Geological Survey Director, and other directors from the different U.S. agencies, who are tasked to develop the National Earthquake Hazards Reduction Program. Apart from interagency cooperation, the U.S. entrusts the U.S. Geological Survey to identify risks of earthquakes through assessments conducted on regions of the U.S., subsequently establishing monitoring projects and conducting studies. Based on such assessments, the agency will develop standard procedures to issue earthquake predictions (*Earthquake hazards reduction act* 1977, as amended 2022).

On the same focus on earthquakes, the U.S. government requires the U.S. Geological Survey in 2018 to establish EWS through the reauthorisation of the National Earthquake Hazards Reduction Program (Rowan 2022). The U.S. Geological Survey relied on ShakeAlert which has long been developed and is now implemented in the West Coast of the U.S. The ShakeAlert detects earthquakes and alerts the public about such earthquakes. Warnings from the U.S. EWS will be sent by FEMA through phone applications to provide citizens enough time to anticipate earthquakes. Regardless, some earthquakes were missed or miscalculated because of the lack of station coverage (Rowan 2022).

Finally, the U.S. also provides relief to citizens post-disasters as part of the *Stafford Act*. Regardless of such relief to be optional, past precedents reflected that the government is willing to provide relief especially in the form of housing towards citizens affected by disasters. *La Union* (La Union Del Pueblo Entero v. FEMA 2010) and *Harvest Family Church* (Harvest Family Church v. FEMA 2017), despite indicating that there may be several critics to post-disaster house reliefs, prove that such reliefs have been provided by the U.S. Government.

#### Implications of failure to conduct effective disaster-management response in the U.S.

As the *Stafford Act* explicitly stated, the responsibility to alleviate citizens’ sufferings during times of disasters is given to the U.S. government. The government is responsible for revising programs related to disaster relief, promoting the development of disaster preparedness and assistance plans, urging the development of hazard mitigation measures, and providing Federal assistance for citizens during disasters (*Stafford Act* 2021).

When agencies fail to fulfil their responsibilities in disaster management, citizens may file a suit in accordance with the *Administrative Procedure Act* (*Administrative Procedure Act* 1946, as amended, 2019), basing their arguments on the *Stafford Act*. The outcome of the suit may vary – ranging from the publication of documents used to determine disaster reliefs, to the reconsideration of disaster relief applications – but outcomes ruling in favour of citizens essentially oblige agencies to fulfil their responsibility to apply ‘ascertainable standards’ in providing citizens with disaster reliefs (La Union Del Pueblo Entero v. FEMA 2010).

*La Union* portrays U.S. citizens’ success in demanding for FEMA’s transparency in providing reliefs. *In casu*, FEMA’s provision of disaster reliefs is not mandatory; however, FEMA is bound to non-discriminatory rules under the *Stafford Act*. Fundamentally, FEMA made available housing reliefs including home repairs because of Hurricane Dolly but denied numerous citizens’ applications under the basis that there have been insufficient damages. Plaintiffs, who were citizens, filed a suit alleging FEMA of violating the non-discriminatory rule. The court ruled that FEMA must publish the standards they used in determining disaster relief applications (La Union Del Pueblo Entero v. FEMA 2010).

*Saint Bernard Parish Government* further exemplifies the possibility of citizens filing a suit against the U.S. government for inaction; the government was accused of failing to prevent the aggravation of disasters. *In casu*, Judge Braden ruled that the U.S. government through the Army Corps of Engineers failed to preserve the Mississippi River-Gulf Outlet (‘*MR-GO*’), intensifying damages caused by the flood post-Hurricane Katrina (St. Bernard Parish Government et al., v. the United States 2018). Later, the ruling was reversed as the U.S. government has made efforts allowing Plaintiffs to be placed in a better position in comparison to if the government did not take any actions (St. Bernard Parish Government et al. v. United States 2018).

Despite the possibility of citizens filing a suit, Section 305 of the *Stafford Act* explicitly provides that the Federal Government is not liable for claims against the discretionary power of Federal agencies in implementing the act. The act thus allows FEMA to be immune from claims addressing FEMA’s discretionary power to provide disaster relief (Barbosa 2019, Petitioners v. Department of Homeland Security). *Saint Bernard Parish Government* further highlighted the government’s inability to be held liable for inaction or failure to act (St. Bernard Parish Government et al. v. United States 2018).

Conclusively, where the U.S. government is considered by citizens to fail in providing adequate disaster relief, the U.S. government and federal agencies are immune to such claims as enshrined under the *Stafford Act*. Citizens, however, can still try to argue under Section 308 of the *Stafford Act* regarding ‘Nondiscrimination in Disaster Assistance’ for claims related to discrimination in the provision of disaster reliefs as exemplified in *La Union*. Such claims include cases where citizens consider that the FEMA is deemed to lack transparency or implement ambiguous standards.

## Conclusion

### Government’s disaster-management duties

To conclude, it is the government’s duty towards its citizens to fulfil citizens’ human rights in the event of natural disasters. Although not all governments expressly require it, the concept of state responsibility, particularly in the event of natural disasters, is largely owned by countries such as the United States and Indonesia. With regard to liability, only strict liability and negligence are appropriate to be contextualised with natural disasters among the numerous categories of liability that exist. Its simple evidentiary properties enable citizens to pursue inclusive justice.

### Evaluating disaster-management practices in Indonesia and the U.S.

Reflecting on the practice of disaster management in both Indonesia and the U.S., the authors will first consider the Indonesian disaster-management practice. First of all, the U.S. and Indonesia have different characteristics of natural disasters. Disaster in the U.S. is dominated by hurricanes and floods. This type of disaster greatly influences the disaster-management paradigm regulated in the main regulations regarding disaster management in the U.S., namely the *Stafford Act*, where hurricane and flood disasters are regulated in a more detailed and comprehensive manner. In its development, the U.S. also has complementary regulations regarding the management of earth disasters through the *Earthquake Hazards Reduction Act* 1977. In Indonesia, geological disasters are a distinctive characteristic and often occur because of Indonesia’s location in a very tectonically active zone. A series of geological disasters starting from the 2004 Aceh Earthquake and Tsunami, the 2005 Nias Earthquake and Tsunami, to the 2006 Yogyakarta Earthquake became the background for the formation of the main regulations in disaster management in Indonesia.

Indonesia has made rapid progress since 2007 with the Indonesian Disaster Management Law which provides the basis that the government is immediately responsible for disaster management. Accordingly, the disaster-management aspects that Indonesia have carried out considerably well include the mandatory obligation of the government to help citizens amidst disasters and the disaster reliefs provided under the aforesaid law. Under the aforementioned law, the various disaster reliefs that start from health-related needs to even compensation for deaths may be considered to cater to the needs of citizens during and post disasters.

Regardless of the strengths listed earlier, Indonesia can still improve its disaster-management system especially in the EWS, disaster communication, interagency cooperation, and budgeting fields. Regarding EWS and disaster communication, the Indonesian InfoBMKG application is still under the Beta version. Moreover, existing buoys are not functioning properly. In the fields of interagency cooperation, Indonesia lacks a systematised system and a line of communication which may hamper effective communication. Noting the decentralisation in Indonesia, a structured and communicative interagency cooperation is the key to a reliable disaster-management system. Finally, the lack of budget allocation remains a problem which hampers the well-constructed disaster-management system in the country.

Conversely, in the U.S., several aspects of disaster management which have been carried out well include the systematised interagency cooperation as reflected by the incident command system. From the U.S. it can be inferred that decentralisation of responsibilities may not be a problem to disaster-management systems so long as interagency cooperation remains effective. Furthermore, the specificity of available assistance and the extent to which they were provided reflect an accommodating post-disaster assistance provided by the government. Finally, the fast circulation of disaster information and EWS reflects a well-executed disaster response mechanism.

Despite the aforementioned strengths, the main drawback is that disaster-management assistance is not compulsory to be provided by the government. Moreover, U.S. agencies are essentially immune from numerous liabilities except if they directly contributed to the occurrence of disasters (St. Bernard Parish Government et al. v. United States 2018) and if they acted in a discriminatory manner when providing disaster reliefs (La Union Del Pueblo Entero v. FEMA 2010). Lastly, despite the speed of the EWS in the U.S., the lack of station coverage would certainly cause several earthquakes to be missed (Rowan 2022).

### Recommendations to improve Indonesia’s disaster-management system based on the best practices of the U.S.

Firstly, accountability and transparency within disaster agencies will facilitate effective disaster- management responses conducted by these agencies. In disseminating early warnings as well as post-disaster treatment ranging from evacuation to aid distribution, accountability, transparency, and agencies’ willingness to cooperate play a crucial role (Boin & Lodge 2016). Secondly, transparency and accountability are crucial aspects of the effectiveness of agencies. In budget audits of disaster agencies, transparency makes it easier for the government to understand the needs of disaster agencies to provide disaster-mitigation services and allocate disaster-related budgets (Tanesab 2020). In the U.S., transparency between disaster agencies is a key factor in minimising overlap in disaster information. On the other hand, Indonesia has not been able to implement the principles of transparency and accountability in disaster management optimally. This can be seen from a series of failures in disaster management caused by poor transparency between disaster agencies, for example in the management of the EWS and processing of disaster data resulting in overlapping information and inaccurate delivery of information to the public, as reflected in the 2018 Sunda Strait Tsunami. From the explanation given earlier, transparency between disaster agencies and related parties will affect disaster agencies’ capabilities in implementing DRR efforts. Therefore, transparency and accountability in disaster management in Indonesia must be improved to create effective disaster management so as to reduce the impacts of disasters.

In regards to EWS, Indonesia needs to further develop the InfoBMKG application as the current available version is the Beta version, which indicates that it is still prematurely developed. This is in contrast to the ShakeAlert application that is available in the U.S. Furthermore, the dysfunctional buoys call for budget allocation and the willingness of disaster management-related agencies to maintain the buoys, understanding that they play a crucial part in EWS to detect upcoming Tsunamis – a disaster that might be considered a regular occurrence in Indonesia (Ahmad 2022).

Taking the U.S. as an example of a best practice, Indonesia may implement a common and universal structure similar to the incident command system on interagency cooperation; the measure may prevent overlapping authorities. The systematised interagency cooperation in the U.S. is also because of the FEMA being appointed as the main authority in cases of disasters and is given the power to coordinate with agencies. Despite BNPB being able to coordinate with agencies, interagency cooperation can still be improved and promoted in Indonesia. When agencies cooperate with a structured system and clarity on the leading institution, overlapping authorities can be prevented, allowing effective interagency cooperation and disaster-management system pre-disaster, during disasters, and post-disaster.

Both the U.S. and Indonesia have provided relief to citizens post-disasters. In the U.S., community aid is part of the *Stafford Act* and is discretionary by the U.S. President. Disaster relief is provided to communities affected by disasters, both during times of crisis such as food, health, and accessibility assistance, and after disasters such as relief in housing to residents affected by disasters. Meanwhile in Indonesia, assistance for affected communities is generally regulated by the Indonesia Disaster Management Law, which mandates the Government to fulfil all the needs of affected residents both during and after a disaster. Despite the existence of several disaster reliefs in Indonesia, the government may consider the provision of specific assistance, that is, the installation of debris traps or even the relocation of persons. Additionally, as temporary housing units have been implemented as part of disaster reliefs in the country, the Indonesian Disaster Management Law can further specify the provision of temporary housing units as part of disaster relief to further concretise the relief.
